# P098 When dryness comes last: recurrent audio vestibular ischemia revealing primary Sjögren’s disease

**DOI:** 10.1093/rap/rkag113.128

**Published:** 2026-11-03

**Authors:** Tejaswee Banavathu, Prasan Deep Rath, Swetal Chouhan, Prakshi Tyagi

**Affiliations:** Max Super Speciality Hospital, Saket, New Delhi, India; Max Super Speciality Hospital, Saket, New Delhi, India; Max Super Speciality Hospital, Saket, New Delhi, India; Max Super Speciality Hospital, Saket, New Delhi, India

## Abstract

**Introduction:**

Primary Sjögren’s disease (pSS) is a systemic autoimmune disorder with neurological involvement in a minority of patients. Central nervous system (CNS) vasculitis is an uncommon but potentially disabling manifestation and may precede classical sicca symptoms, resulting in delayed diagnosis. Recurrent posterior circulation audio vestibular syndromes are usually attributed to cerebrovascular disease, while autoimmune causes are rarely considered. We report a patient with sequential sensorineural hearing loss, recurrent posterior circulation ischaemic events and occult sicca symptoms, in whom targeted rheumatological assessment, salivary gland ultrasonography and intracranial vessel-wall magnetic resonance imaging established the diagnosis of pSS-associated CNS vasculitis.

**Case description:**

A 51-year-old man presented with sudden-onset severe vertigo, persistent vomiting, gait imbalance and simultaneous painless complete hearing loss in the left ear for three days. Four months earlier, he had experienced an acute episode of vertigo with right upper-limb clumsiness and was diagnosed with a right anterior inferior cerebellar artery territory infarction. Five years before presentation, he had developed sudden irreversible right-sided sensorineural hearing loss. Despite dual antiplatelet therapy and statin treatment, he developed recurrent posterior circulation symptoms.

Neurological examination demonstrated bilateral sensorineural hearing loss, gaze-evoked nystagmus, truncal ataxia and dysmetria without focal motor or sensory deficits. Inflammatory markers were elevated (ESR 43 mm/hour, CRP 40 mg/L). Extensive evaluation for conventional stroke mechanisms, including thrombophilia and antiphospholipid antibodies, was unrevealing. Digital subtraction angiography showed posterior circulation vessel irregularities with a small proximal posterior inferior cerebellar artery aneurysm, while cerebrospinal fluid revealed mildly elevated protein without infection or demyelination.

During rheumatology review, a directed history uncovered a three-year history of xerostomia requiring frequent water intake with meals, associated with progressive dental caries and tooth loss, despite absent ocular dryness or other connective tissue disease manifestations. Examination demonstrated poor dentition and reduced salivary pooling. Autoimmune evaluation revealed strongly positive antinuclear antibodies (>1:2560, SSA pattern) with anti-Ro60 and anti-Ro52 positivity. Salivary gland ultrasonography demonstrated grade 2 bilateral parotid and submandibular gland abnormalities with grade 1 lacrimal gland involvement. High-resolution intracranial vessel-wall magnetic resonance imaging demonstrated concentric enhancement of both petrous internal carotid arteries, favouring inflammatory vasculitis.

A diagnosis of primary Sjögren’s disease with CNS vasculitis (ESSDAI 15) was established. The patient received intravenous methylprednisolone followed by six monthly cycles of cyclophosphamide and maintenance mycophenolate mofetil. Vertigo improved substantially, partial hearing recovery occurred, and no further ischaemic events developed during follow-up.

**Discussion:**

Neurological manifestations precede sicca symptoms in a substantial proportion of patients with primary Sjögren’s disease, making diagnosis particularly challenging. This patient initially fulfilled a conventional stroke pathway because of recurrent posterior circulation ischaemic events and sequential audio vestibular involvement. However, the recurrence despite optimal secondary stroke prevention and the absence of an identifiable thromboembolic, atherosclerotic or prothrombotic cause prompted reconsideration of the diagnosis.

The pivotal diagnostic clue emerged from a targeted rheumatological history rather than laboratory investigations. Chronic xerostomia with progressive dental destruction, initially considered insignificant by the patient, redirected evaluation towards an autoimmune aetiology. Positive anti-Ro antibodies, characteristic salivary gland ultrasonography findings and high-resolution intracranial vessel-wall magnetic resonance imaging collectively supported inflammatory vasculopathy secondary to primary Sjögren’s disease.

This case also highlights the evolving role of vessel-wall magnetic resonance imaging in differentiating inflammatory vasculitis from intracranial atherosclerosis, reversible cerebral vasoconstriction syndrome and embolic disease. Demonstration of concentric vessel-wall enhancement substantially increased diagnostic confidence and supported escalation to aggressive immunosuppressive therapy despite non-specific conventional neuroimaging.

Prompt recognition altered management completely. Rather than intensifying antithrombotic therapy, the patient received immunosuppression with glucocorticoids, cyclophosphamide and maintenance mycophenolate mofetil, resulting in neurological stabilisation and prevention of further cerebrovascular events.

Beyond illustrating a rare manifestation of primary Sjögren’s disease, this case reinforces an important clinical principle: recurrent audio vestibular ischaemic syndromes should not automatically be attributed to conventional cerebrovascular disease. Careful reassessment, directed questioning for subtle sicca symptoms and judicious use of contemporary rheumatological imaging may reveal a treatable inflammatory disorder before irreversible neurological disability occurs.

**Key learning points:**

• Primary Sjögren’s disease should be considered in patients with unexplained recurrent posterior circulation neurological syndromes, even when classical sicca symptoms are absent or appear trivial.

• Neurological manifestations may precede the diagnosis of primary Sjögren’s disease by several years. Sequential sensorineural hearing loss, recurrent vertigo and cerebellar ischaemic events may represent autoimmune vasculopathy rather than conventional cerebrovascular disease.

• Repeated negative investigations for common stroke mechanisms should prompt diagnostic re-evaluation rather than repeated attribution to cryptogenic stroke.

• A meticulous rheumatological history remains one of the most valuable diagnostic tools. Directed questioning regarding xerostomia, increased water intake during meals, progressive dental caries and tooth loss may uncover clinically significant sicca that patients fail to volunteer spontaneously.

• Salivary gland ultrasonography provides rapid, non-invasive evidence of glandular involvement and complements serological evaluation. High-resolution intracranial vessel-wall magnetic resonance imaging is an emerging technique that improves diagnostic confidence by distinguishing inflammatory vasculitis from atherosclerotic and other intracranial vasculopathies.

• Early recognition of autoimmune CNS vasculitis is essential because management differs fundamentally from conventional secondary stroke prevention. Timely initiation of immunosuppressive therapy can stabilise neurological disease, prevent recurrent ischaemic events and reduce irreversible disability.

• This case emphasises the value of multidisciplinary collaboration between neurologists, neuroradiologists and rheumatologists in solving diagnostically challenging neuro-immunological presentations.

